# Resistance of hypervirulent *Klebsiella pneumoniae* to cathepsin B-mediated pyroptosis in murine macrophages

**DOI:** 10.3389/fimmu.2023.1207121

**Published:** 2023-06-29

**Authors:** Jin Kyung Kim, Hui-Jung Jung, Miri Hyun, Ji Yeon Lee, Jong-Hwan Park, Seong-Il Suh, Won-Ki Baek, Hyun ah Kim

**Affiliations:** ^1^ Department of Microbiology, Keimyung University School of Medicine, Daegu, Republic of Korea; ^2^ Department of Infectious Diseases, Keimyung University Dongsan Hospital, Keimyung University School of Medicine, Daegu, Republic of Korea; ^3^ Laboratory Animal Medicine, College of Veterinary Medicine and Brain Korea 21 Plus Project Team, Chonnam National University, Gwangju, Republic of Korea

**Keywords:** hypervirulent, *Klebsiella pneumoniae*, macrophages, pyroptosis, cathepsin B

## Abstract

**Introduction:**

Hypervirulent *Klebsiella pneumoniae* (hvKp) has emerged as a clinically significant global pathogen in the last decade. However, the host immune responses of the macrophages during hvKp infection are largely unknown. In the present study, we aimed to compare the cytotoxic effects of hvKp and classical *K. pneumoniae* (cKp) in murine macrophages.

**Results:**

We found that the activation of caspase-1 -dependent pyroptosis was higher in cKp-infected macrophages compared with that in hvKp-infected macrophages. In *Caspase-1* deficiency macrophages, pyroptosis diminished during infection. Both hvKp and cKp strains led to nucleotide-binding and oligomerization domain-like receptor protein 3 (NLRP3) inflammasome formation and lysosomal cathepsin B activation, thus resulting in pyroptosis. Compared with the cKp strain, the hvKp strain inhibited these phenomena in murine macrophages.

**Conclusion:**

HvKp infection resulted in different levels of pyroptosis via the activation of cathepsin B-NLRP3-caspase-1 in murine macrophages. Therefore, the manipulation of pyroptotic cell death is a potential target for host response during hvKp infection in macrophages.

## 1 Introduction

Classical *Klebsiella pneumoniae* (cKp) usually presents as hospital-acquired pneumonia, urinary tract infection, and bacteremia in an immunocompromised host (1). It is a “critical concern” of the World Health Organization because of its carbapenem resistance (2). In contrast, hypervirulent *K. pneumoniae* (hvKp), which usually presents as community liver abscess, has emerged in East Asian countries in the last three decades. Usually, hvKp causes liver abscess, endophthalmitis, meningitis, and necrotizing fasciitis in healthy individuals with community origin (3). Recently, hvKp has spread globally and the prevalence of multidrug-resistant hvKp also increased (4, 5). Therefore, to respond to hvKp, we need to increase awareness of the pathogenesis of this strain. Metastatic infection due to hvKp is the hallmark clinical feature and it occurs through neutrophils (6). Additionally, hvKp is involved in cell death of neutrophils (6), but the mechanism has not been clearly identified.

Cell death is an important physiological process that plays a pivotal role in maintaining homeostasis in living organisms. Three types of cell death (pyroptosis, apoptosis, and necroptosis) have been well known and studied (7). Particularly, pyroptosis, called inflammatory cell death, is induced by inflammasome sensors such as the nucleotide-binding oligomerization domain-like receptor family, absent in myeloma 2 (AIM2), and pyrin receptor (8). In the canonical pyroptosis pathway, the inflammasome is induced by danger-associated molecular patterns and pathogen-associated molecular patterns. Consequently, pro-interleukin (IL)-1β, IL-18, and gasdermin D (GSDMD) are cleaved via activated caspase-1. The N-terminal from cleaved GSDMD forms pores in the plasma membrane, resulting in the secretion of IL-1β and IL-18 into the extracellular space (9). This pyroptosis is related to diverse human diseases, such as tumor (9), liver fibrosis (10), cardiovascular disease (11), and central nervous system disease (12).

Lysosomal cysteine cathepsins (B, C, F, H, K, L, O, S, V, Z, and W) are involved in nucleotide-binding and oligomerization domain-like receptor protein 3 (NLRP3) inflammasome activation (13). Among them, cathepsin B can interact with NLRP3 upon inflammasome activator treatment (14); thus, it activates the NLRP3 inflammasome under diverse stimuli (15, 16). Several studies have shown that pyroptosis is triggered by cathepsin B-mediated NLRP3 inflammasome activation (17, 18). Wang et al. suggested that coxsackievirus B3 infection activates cathepsin B and promotes pyroptosis (19); however, the crosstalk between cathepsin B and pyroptosis during pathogen infection is largely unknown.

Herein, we found that hvKp infection exhibited weakened pyroptosis compared to the cKp strain. Mechanistically, cathepsin B-mediated NLRP3 activation was significantly higher in cKp*-*infected macrophages than in hvKp-infected macrophages. In addition, *Caspase-1*-null macrophages showed reduced pyroptosis during cKp infection compared with wild-type cells.

## 2 Materials and methods

### 2.1 Ethics statements

This study was approved by the Institutional Research and Ethics Committee at Keimyung University (approval number: KM-2022-07R1). All animal experiments were performed in accordance with the guidelines of the Korean Food and Drug Administration.

### 2.2 Mice

Wild-type (WT) C57BL/6 female mice (age; 7–9 weeks, weight; 20–25 g) were purchased from Samtako BioKorea Co. (Osan, Korea). *Caspase-1* ^-/-^ female mice (age; 7–9 weeks, 20-25 g) were kindly provided by Prof. Gabriel Núñez (University of Michigan Medical School, Ann Arbor, MI, USA) (20). Mice were maintained under a 12-h light/dark cycle at an ambient temperature of 20–23°C and relative humidity of 40–60% and with ample food and water. All animals were maintained under barrier conditions in a biohazard animal room at the School of Medicine, Keimyung University, Daegu, Korea.

### 2.3 Cell culture

The murine macrophage cell lines RAW264.7 (American Type Culture Collection, TIB-71) were maintained in the Dulbecco’s Modified Eagle’s Medium (DMEM; Welgene, Gyeonsan, South Korea), supplemented with 10% fetal bovine serum (Welgene) and 1% penicillin/streptomycin (Gibco BRL, Grand Island, NY, USA). Cells were incubated in a humidified atmosphere at 37°C with 5% CO_2_. Primary bone marrow-derived macrophages (BMDMs) were isolated from C57BL/6 mice and cultured in DMEM for 3–5 days in the presence of macrophage colony-stimulating factor (M-CSF; R&D Systems, Minneapolis, MN, USA).

### 2.4 Bacterial strains and culture

Sixteen Kp isolates were obtained from the various clinical specimens of Keimyung University Dongsan Hospital. From these strains, we collected the hvKp strain from a blood specimen of a patient with a liver abscess, whereas cKp was collected from the sputum of a patient with hospital-acquired pneumonia. These isolates were identified using the automated microbial identification and susceptibility test system (VITEK 2 system, bioMerieux, Lyon, France). Extended-Spectrum ß-Lactamase (ESBL) production was confirmed by agar dilution test using cefotaxime and ceftazidime along with clavulanate, in accordance with the Clinical and Laboratory Standards Institute guidelines.

Capsular serotypes and genes of virulence factors were identified by polymerase chain reaction (PCR) (21, 22). Strains were serotyped as: K1, K2, K5, K20, K54, and K57, or as non-determining when no specific serotype could be identified. Hypervirulent characteristics were confirmed with a string test and gene amplification of *rmpA*, *magA*, and aerobactin. According to serotype analysis, the hvKp was a K1 serotype. HvKp had antibiotic susceptibility for most antibiotics except ampicillin. The cKp strain had a negative string test result, and, according to genetic analysis, it was negative for *rmpA*, *magA*, and aerobactin. The antibiotic susceptibility test result was positive for ESBL and showed resistance for most antibiotics except carbapenem and tigecycline.

Strains of Kp were grown in Luria-Bertani (LB) broth (Difco, Becton Dickinson, Sparks, MD, USA) for liquid culture and on MacConkey agar (Difco) as a solid medium at 37°C. All bacteria were stored at -80°C in LB broth supplemented with 15% v/v glycerol (Sigma-Aldrich, St. Louis, MO, USA). For infection stocks, bacteria were incubated overnight and then diluted in fresh LB broth and cultured to an optical density (OD 600 nm) of 0.1. Samples were frozen at -80°C with 15% glycerol, and one aliquot was counted prior to infection.

### 2.5 Infection

BMDMs were replated into 24- or 96-well plates in complete DMEM medium at a density of 2 × 10^5^ cells/well and infected with the different strains at a multiplicity of infection (MOI) of 10 for 90 min. RAW264.7 cells were replated into 6-well or 96-well plates in complete DMEM medium at a density of 7 × 10^5^ cells/well and infected with the different strains at MOI of 10 for 90 min. Cells were washed twice with phosphate-buffered saline (PBS) containing antibiotics (gentamicin 100 μg/ml) to remove non-phagocytosed bacteria.

### 2.6 Treatments

#### 2.6.1 Inhibition of cathepsin B

CA-074 methyl ester (CA074-Me; S7420; Selleck Chemical, Houston, TX, USA) is a specific inhibitor of cathepsin B. To inhibit cathepsin B activity, CA074-Me was dissolved in dimethylsulfoxide (DMSO) at a concentration of 50 mM. Cells were treated in media containing 25 μM CA074-Me for 1 h prior to bacterial infection. BMDMs and RAW264.7 cells were infected with hvKp or cKp strains at MOI of 10 for 90 min, washed and then cultivated for 16 h.

#### 2.6.2 Inhibition of caspase-1

Ac-YVAD-cmk was manufactured by the Sigma-Aldrich (SML0429; St. Louis, MO, USA) and solubilized in DMSO (stock 50 mM). Cells were grown in media containing 50 μM Ac-YVAD-cmk or an equivalent amount of vehicle (DMSO). Cells were treated in media containing 50 μM Ac-YVAD-cmk for 30 min prior to bacterial infection. 

### 2.7 Immunoblotting

Cells were lysed in RIPA lysis buffer (20 mM Tris-HCl, pH 7.4, 137 mM NaCl, 10% glycerol, 1% Triton X-100, 1 mM Na3VO4, 1 mM NaF, 2 mM ethylenediaminetetraacetic acid [EDTA], 200 nM aprotinin, 20 μM leupeptin, 50 mM phenanthroline, and 280 mM benzamidine-HCl) and supernatant fractions were collected. Protein concentrations were measured with bicinchoninic acid (Pierce, Rockford, IL, USA), and equal amounts of proteins (50 μg/lane) were separated by 13% sodium dodecyl-sulfate polyacrylamide gel electrophoresis and transferred to the nitrocellulose membranes (GE Healthcare Life Science, Pittsburgh, PA, USA) incubated with a specific antibody. The anti-caspase-1 antibody (3019-100; BioVision, Milpitas, CA, USA, and AG-20B-0042; AdipoGen, San Diego, CA, USA) and anti-GSDMD antibody (ab209845; Abcam PLC) were used as primary antibodies. These antibodies were used at a dilution of 1:1,000. The anti−ACTB antibody (A5441) was acquired from Sigma−Aldrich (Merck KGaA) and used at a dilution of 1:5000. Secondary antibodies (anti−rabbit IgG [1:2000 diluted; sc−2004] and anti−mouse IgG [1:2000 diluted; sc−2005]) were purchased from Santa Cruz Biotechnology, Inc. (Dallas, TX, USA). Bands were detected using the Immobilon Western Chemiluminescent HRP Substrate (EMD Millipore, Darmstadt, Germany).

### 2.8 RNA isolation and quantitative real-time polymerase chain reaction (qRT-PCR)

Total RNA was extracted from infected cells using TRIzol (15596-026; Thermo Fisher Scientific, Waltham, MA, USA), following the manufacturer’s instructions. Then, RNA was reverse transcribed to complementary DNA using the reverse transcriptase premix (Elpis Biotech, EBT-1515; Elpis Biotech, Lexington, MA, USA). Data were analyzed by the qTOWER^3^ PCR thermal cycler (Analytik Jena, Jena, Germany) using the TOPreal qPCR 2X SYBR Green PreMIX (Enzynomics, RT500M). The gene expression was calculated using the 2^−ΔΔCt^ method and normalized to *b-actin*. The primer sequences (mouse) were as follows: *Il1b* forward: 5’- TACGGACCCCAAAAGATGA-3’, reverse: 5’-TGCTGCTGCGAGATTTGAAG-3’; *Il18* forward: 5’-GACAGCCTGTGTTCGAGGATATG-3’, reverse: 5’-TGTTCTTACAGGAGAGGGTAGAC-3’; *Nlrp3* forward: 5’-TCACAACTCGCCCAAGGAGGAA-3’, reverse: 5’-AAGAGACCACGGCAGAAGCTAG-3’; and *b-actin* forward: 5’- CCACCATGTACCCAGGCATT-3’, reverse: 5’- AGGGTGTAAAACGCAGCTCA-3’

### 2.9 Flow cytometric analysis

Cell cycle analysis was performed using flow cytometry. For DNA content analysis, approximately 10^6^ cells were fixed in 80% ethanol for at least 1 h at 4°C. Ethanol-fixed cells were stained with propidium iodide (PI) staining solution (50 μg/ml PI, 0.1 mg/ml RNase A, 0.1% NP-40, 0.1% trisodium citrate) for 30 min, and analyzed using a FACS analyzer (BD Biosciences, San Diego, CA, USA).

### 2.10 Lactate dehydrogenase production assay

Lactate dehydrogenase (LDH) release into the culture supernatant was measured using the EZ-LDH assay kit (DoGenBio, Seoul, Korea), following the manufacturer’s instructions. Briefly, BMDMs or RAW264.7 cells were cultured and infected with hvKp or cKp. Then, the supernatant medium (10 µl) for each sample was transferred into a 96-well plate and was incubated with the LDH reaction mixture (100 µl) at 25°C for 30 min in a dark room. LDH levels in the culture medium were determined by measuring the absorbance (450 nm) using the Synergy/HTX (BioTek instrument, Inc. Winooski, VT, USA).

### 2.11 Enzyme-linked immunosorbent assay (ELISA)

BMDMs and RAW264.7 cells were cultured in 24-well culture plates at a density of 2 × 10^5^/well. Media were collected after overnight culture and centrifuged at 800 rpm for 5 min at 4°C to remove cell debris. Culture supernatants were assayed for IL-1β using the mouse IL-1β/IL-1F2 Quantikine ELISA Kit from R&D Systems (MLB00C, Minneapolis, MN, USA) according to the manufacturer’s instructions.

### 2.12 Immunofluorescence and analysis

For NLRP3 and ASC staining, cells were fixed with 4% paraformaldehyde for 15 min and then permeabilized with 0.25% Triton X-100 (Sigma-Aldrich) for 10 min. Next, cells were incubated with NLRP3 (1:400 diluted; 15101; Cell Signaling, Danvers, MA, USA), ASC (1:400 diluted; sc-514414; Santa Cruz Biotechnology, Inc.), and cleaved GSDMD (1:400 diluted; 34667; Cell Signaling, Danvers, MA, USA) primary antibodies overnight at 4°C. Then, cells were washed with PBS followed by an incubation with secondary Alexa Fluor 488 goat anti-rabbit IgG (H+L) (1:400 diluted; A-11034, Invitrogen) and Donkey anti-mouse IgG (H+L) Alexa Fluor Plus 594 (1:400 diluted; A32744, Invitrogen) at room temperature for 2 h. Nuclei were stained with 4′,6-diamidino-2-phenylindole (D9542; Sigma-Aldrich) for 5 min at room temperature. The infected cells were stained with Lysotracker Yellow-HCK-123 (L12491; Thermo Fisher Scientific), according to the manufacturer’s instructions. Immunofluorescence images were observed and analyzed using the confocal laser microscope (TCS SP8; Leica Microsystems, Wetzlar, Germany) and Image J software (National Institutes of Health, Bethesda, MD, USA).

### 2.13 Caspase-1 activity assay

Caspase-1 activity was measured using the FAM-FLICA Caspase-1 (YVAD) Assay Kit (#98; ImmunoChemistry Technologies, Davis, CA, USA) according to the manufacturer’s instructions. Murine macrophages on coverslips were infected and stained with a staining solution at 37°C for 1 h. After washing with 1× apoptosis wash buffer, cells were stained with Hoechst 33342 at 37°C for 10 min and fixed with a fixative. The samples were acquired and analyzed with a confocal laser microscope (TCS SP8; Leica Microsystems, Wetzlar, Germany) and Image J software.

### 2.14 Detection of cathepsin B activation

The infected cells were stained using the Magic Red Cathepsin B assay kit (#938; ImmunoChemistry Technologies) at 37°C for 1 h and nuclei were stained with Hoechst 33342 at 37°C for 10 min. Cells were fixed with 4% paraformaldehyde and images were visualized by a confocal laser microscope (TCS SP8; Leica Microsystems, Wetzlar, Germany).

### 2.15 Statistical analysis

Data are presented as mean ± standard deviation and were obtained from three independent experiments. The Student t-test or analysis of variance was used to analyze the data. Statistical analyses were performed, and graphs were made using GraphPad Prism 7.0 software for Windows (GraphPad Software Inc., La Jolla, CA, USA). P-values <0.05, 0.01, and 0.001 were considered statistically significant.

## 3 Results

### 3.1 The hvKp infection suppresses the GSDMD-derived pyroptosis in BMDMs compared with cKp infection

**Figure 1:**
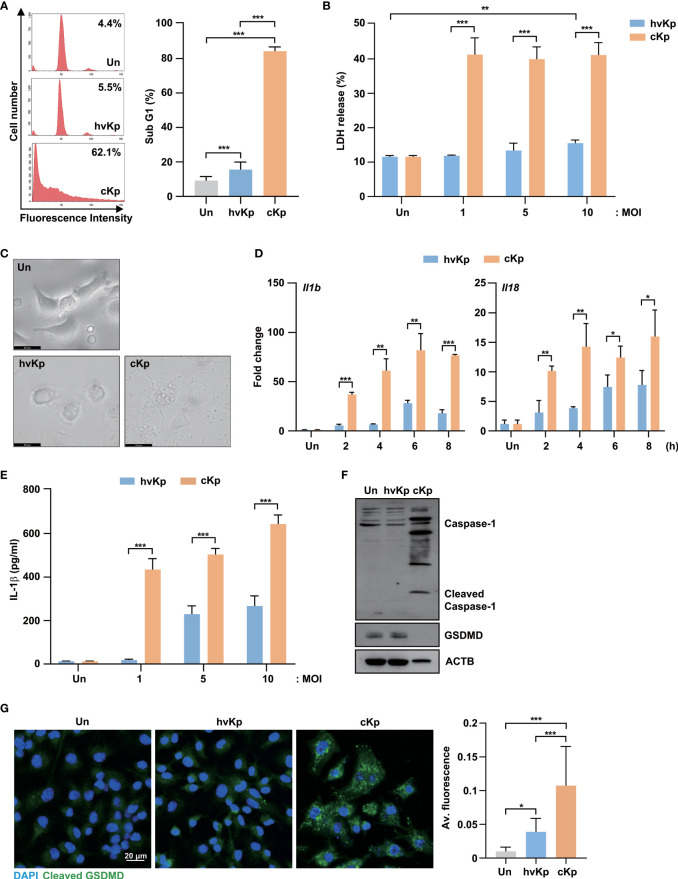
HvKp and cKp strains induce pyroptotic cell death in BMDMs. **(A–C)** BMDMs were infected with hvKp or cKp (MOI of 10) for 16 h. Cell death was analyzed using **(A)** flow cytometry with propidium iodide (PI) staining and **(B)** LDH release in the supernatant. The number of PI+ macrophages was determined using flow cytometry and is shown by a representative value from the three independent experiments. **(C)** The cell morphologies were determined by bright-field microscopy. Images were magnified ×400. Scale bar, 20 μm. **(D)** BMDMs were infected with hvKp or cKp (MOI of 10) for the indicated times, and the mRNA levels of *Il1b* and *Il18* were measured by qRT-PCR. **(E)** BMDMs were infected with hvKp or cKp at different MOI for 16 h. The IL-1β levels were determined using ELISA in cell culture medium. **(F)** BMDMs were infected with hvKp or cKp (MOI of 10) for 16 h. Cells were lysed and subjected to immunoblotting using antibodies against caspase-1, GSDMD and ACTB. **(G)** BMDMs were infected with hvKp or cKp (MOI of 10) for 16 h. Cells were stained with cleaved GSDMD (green) and DAPI (for nuclei). Representative images were analyzed using confocal microscopy. Scale bar, 20 μm (left). The quantification of cleaved GSDMD fluorescence (right). Data are expressed as the mean ± standard deviation of the three independent experiments. *P < 0.05, **P < 0.01 and ***P < 0.001 according to analysis of variance. Un, uninfected; Av, average.

To compare the host responses to hvKp and cKp infection within the murine macrophages, clinical hvKp and cKp strains were isolated from blood and sputum samples, respectively (**Supplementary Table 1**). Since a previous study reported that two clinical strains of Kp showed different cell death patterns (23), we investigated whether hvKp and cKp affected the different cell fates in murine macrophages. To examine this, we infected BMDMs with hvKp and cKp strains and measured the sub-G1 population, which represents the apoptotic cells. As shown in **Figure 1A**, cKp infection induced excessive cell death, while hvKp strains exhibited weaker responses in the BMDMs. As expected, hvKp infection induced lower levels of LDH release in an MOI-dependent manner compared with cKp infection (**Figure 1B**). These data indicate that cKp infection triggers severe cell death in BMDMs compared with hvKp infection.

To distinguish the types of cell death, we observed the morphological changes in BMDMs during hvKp and cKp infection using bright-field microscopy. The cKp infection caused severe cell membrane rupture and cell swelling (**Figure 1C**). Therefore, we measured the expression mRNA levels of interleukin (Il)*1b* and *Il18*, which are induced by pyroptosis activation, in BMDMs during infection. Similarly, the cKp-infected cells showed higher mRNA expression levels of *Il1b* and *Il18* compared with hvKp-infected cells (**Figure 1D**). Moreover, ELISA showed the low level of IL-1β production in hvKp-infected BMDMs (**Figure 1E**). During pyroptosis, full-length GSDMD, a pyroptosis-associated protein, was cleaved by inflammatory caspases, and its N-terminus assembled to form pores in the plasma membrane (24). To confirm the occurrence of GSDMD-mediated pyroptosis during hvKp and cKp infection, we measured the GSDMD protein levels. Full-length GSDMD expression level was significantly decreased in cKp-infected cells compared with that in hvKp-infected cells, while the levels of activated caspase-1 increased in the cKp-infected BMDMs (**Figure 1F**). Confocal analysis showed that the expression level of cleaved GSDMD was significantly increased in hvKp- and cKp-infected BMDMs (**Figure 1G**). The cKp infection exhibited higher the fluorescence of cleaved GSDMD when compared to hvKp infection (**Figure 1G**). These results demonstrate that hvKp infection in BMDMs reduces the levels of GSDMD-mediated pyroptosis compared with that during cKp infection.

### 3.2 The cKp infection promotes pyroptosis in RAW264.7 cells compared with hvKp infection

**Figure 2:**
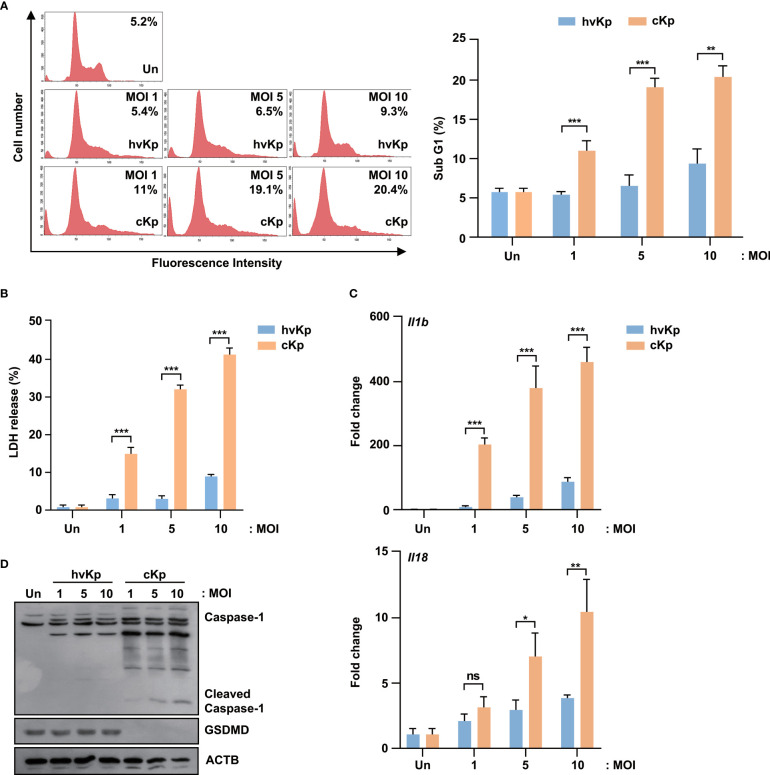
HvKp and cKp strains induce pyroptotic cell death in the RAW264.7 murine macrophage cell line. **(A, B)** RAW264.7 cells were infected with hvKp or cKp (MOI of 1, 5, and 10) for 16 h. Cell death was analyzed using **(A)** flow cytometry with propidium iodide (PI) staining and **(B)** LDH release in the supernatant. The number of PI+ macrophages was determined by flow cytometry and is shown by a representative value from the three independent experiments. **(C)** RAW264.7 cells were infected with hvKp and cKp for 2 h, and the mRNA levels of *Il1b* and *Il18* were measured by qRT-PCR. **(D)** RAW264.7 cells were infected with hvKp or cKp (MOI of 1, 5, and 10). After incubation for 16 h, the cells were lysed and subjected to immunoblotting using antibodies against caspase-1, GSDMD, and ACTB. Data are expressed as the mean ± standard deviation of the three independent experiments. *P < 0.05, **P < 0.01 and ***P < 0.001 according to analysis of variance. Un, uninfected; ns, not significant.

To evaluate the effects of hvKp and cKp infection in the murine macrophage cell line, we infected RAW264.7 cells with hvKp and cKp strains and measured the sub-G1 population. As shown in **Figure 2A**, both strains exhibited an increase in the sub-G1 phase cells in a MOI-dependent manner. Consistent with the BMDMs results, cKp infection increased the percentage of the sub-G1 population in RAW264.7 cells. Similarly, the level of LDH release from cKp-infected RAW264.7 cells was significantly higher than that from hvKp-infected cells at different MOIs (**Figure 2B**). In addition, qRT-PCR analysis showed a higher expression level of *Il1b* and *Il18* in cKp-infected RAW264.7 cells than in hvKp-infected RAW264.7 cells, in a MOI-dependent manner (**Figure 2C**). By contrast, hvKp infection induced relatively low levels of *Il1b* and *Il18* expression in these cells (**Figure 2C**). Like in infected BMDMs, the full-length GSDMD was cleaved, and the level of activated caspase-1 was higher in cKp-infected RAW264.7 cells compared to that observed in hvKp-infected RAW264.7 cells (**Figure 2D**). These data indicate that the level of cKp-induced pyroptosis is higher than that of hvKp-induced pyroptosis in RAW264.7 murine macrophage cell line.

### 3.3 Association between the level of hvKp- and cKp-induced pyroptosis and caspase-1 expression in murine macrophages

**Figure 3:**
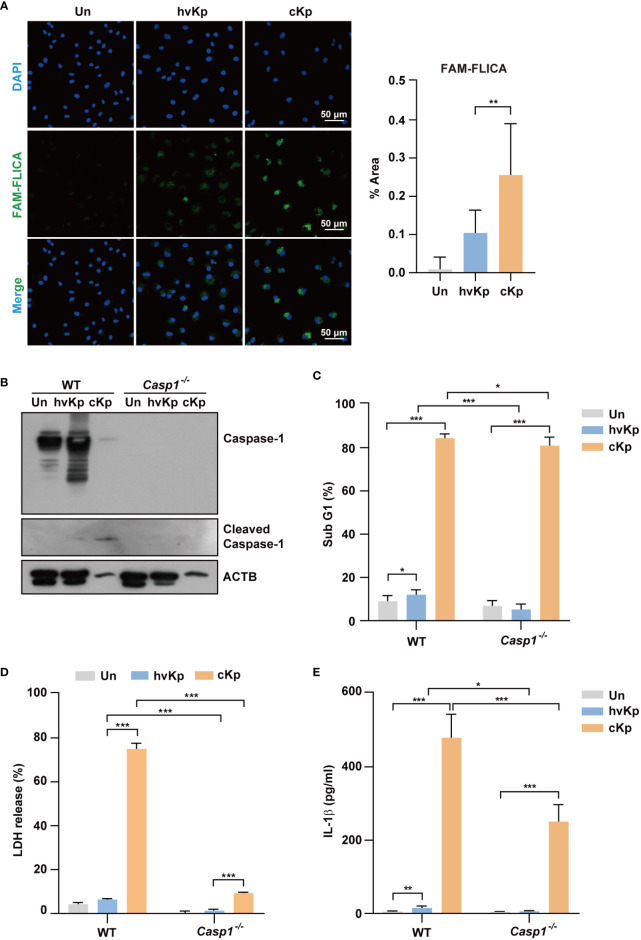
Infection of hvKp and cKp induces caspase-1-dependent pyroptosis in BMDMs. **(A)** BMDMs were infected with hvKp or cKp (MOI of 10) for 8 h, and cells were stained with FAM-FLICA for 1 h. Nuclei were stained with Hoechst 33342. The images were visualized using confocal microscopy. Scale bar, 50 μm (left). Quantification of the FAM-FLICA area (right). **(B–E)** BMDMs were differentiated from WT and *Caspase-1* ^-/-^ mice and infected with the hvKp or cKp strains (MOI of 10) for 16 h. **(B)** Proteins were detected using immunoblotting. Cell death induced by the different strains was determined by **(C)** PI incorporation and **(D)** LDH release in the supernatant. **(E)** The secretion of IL-1β was measured by ELISA. Data are expressed as the mean ± standard deviation of the three independent experiments. *P < 0.05, **P < 0.01 and ***P < 0.001 according to analysis of variance. Un, uninfected; Casp1, caspase-1.

Similar to western blot analysis, confocal microscopy showed that caspase-1 expression level was significantly higher in cKp-infected macrophages compared with that in hvKp-infected macrophages (**Figure 3A** for BMDMs and **Supplementary Figure 1A** for RAW264.7 cells). Therefore, we examined the effects of a caspase-1 inhibitor (Ac-YVAD-cmk) on the production of LDH upon infection. The caspase-1 inhibitor reduced the levels of LDH release from the supernatant of infected macrophages (**Supplementary Figure 1B** for BMDMs and **Supplementary Figure 1C** for RAW264.7 cells), which indicated the involvement and level of caspase-1 activity in pyroptosis.

We further investigated the effects of caspase-1 in the cell death in macrophages from WT and *Caspase-1* ^-/-^ mice. The cleavage of caspase-1 following cKp infection in WT BMDMs was diminished in *Caspase-1* ^-/-^ BMDMs (**Figure 3B**). Furthermore, the cytotoxic effect of cKp infection in *Caspase-1* ^-/-^ BMDMs remarkably reduced compared with that in WT BMDMs (**Figures 3C, D**). In addition, the increased percentage of the sub-G1 population and level of LDH release following hvKp infection in WT BMDMs were inhibited in *Caspase-1* ^-/-^ BMDMs (**Figures 3C, D**). However, the involvement of caspase-1 in the cytotoxicity assays was higher in cKp-infected BMDMs compared with that in hvKp-infected BMDMs. ELISA result showed that the IL-1β production was reduced in *Caspase-1* ^-/-^ BMDMs compared with that in WT BMDMs during infection (**Figure 3E**). Collectively, these data demonstrate that caspase-1 is required for the activation of pyroptosis in macrophages during infection.

### 3.4 The cKp infection promotes the activation of NLRP3 inflammasome compared with hvKp infection

**Figure 4:**
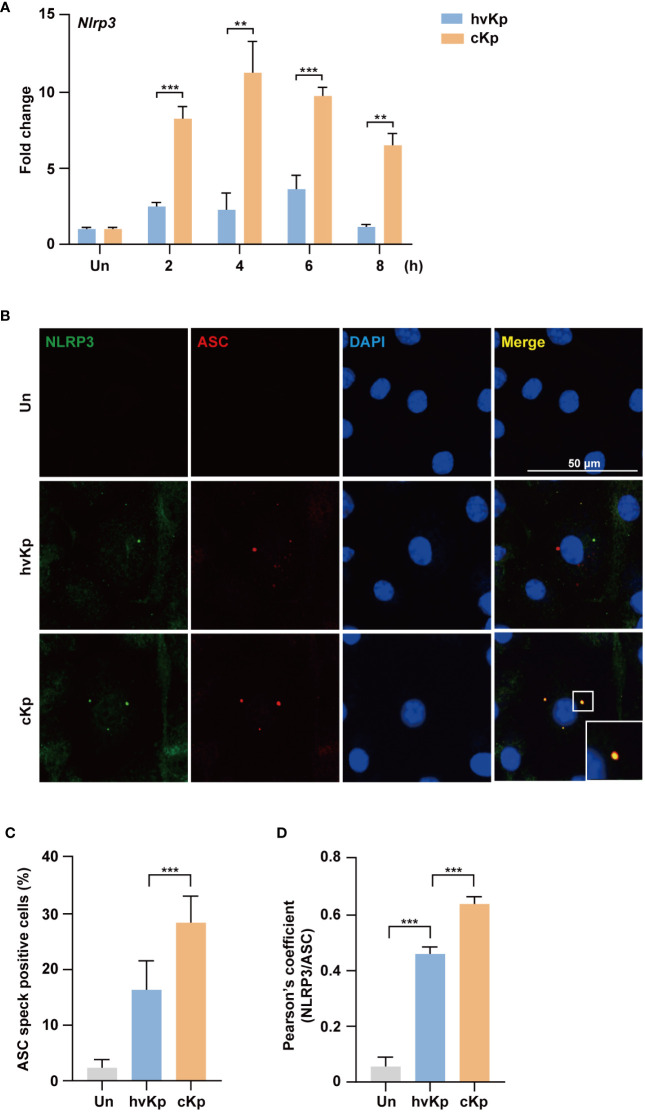
HvKp and cKp infections induce NLRP3 inflammasome formation. **(A)** BMDMs were infected with hvKp or cKp (MOI of 10) for the indicated times and the mRNA level of *Nlrp3* was measured by qRT-PCR. **(B–D)** BMDMs were infected with hvKp or cKp (MOI of 10) for 8 h and cells were stained with NLRP3 (green), ASC (red), and DAPI (for nuclei). **(B)** Representative images were analyzed using confocal microscopy. Scale bar, 50 μm. **(C)** The quantification of ASC speck positive cells. **(D)** NLRP3 and ASC colocalization were analyzed by calculating the Pearson’s correlation coefficient. Data are expressed as the mean ± standard deviation of the three independent experiments. **P < 0.01 and ***P < 0.001 according to analysis of variance. Un, uninfected.

The NLRP3 inflammasome is involved in the induction of pyroptosis during pathogen infection (25–27). Therefore, we explored whether the NLRP3 inflammasome was activated during hvKp and cKp infection. The qRT-PCR analysis showed that hvKp and cKp infection upregulated the mRNA level of *Nlrp3* in BMDMs (**Figure 4A**), which indicates that hvKp and cKp infections enhance the occurrence of pyroptosis via the formation of NLRP3 inflammasome in BMDMs. However, the expression level of *Nlrp3* inflammasome gene was different. As expected, the expression level of *Nlrp3* was significantly lower in hvKp-infected BMDMs (**Figure 4A**). Using confocal microscopy, we confirmed that the number of ASC, an adaptor protein in the NLRP3 inflammasome (28). The percentage of speck positive cells and the colocalization of NLRP3 with ASC were lower after hvKp infection compared with that after cKp infection (**Figures 4B–D**). Therefore, hvKp and cKp infections enhance the occurrence of pyroptosis via the formation of NLRP3 inflammasome in BMDMs.

### 3.5 Involvement of cathepsin B in NLRP3-mediated pyroptosis during infection

**Figure 5:**
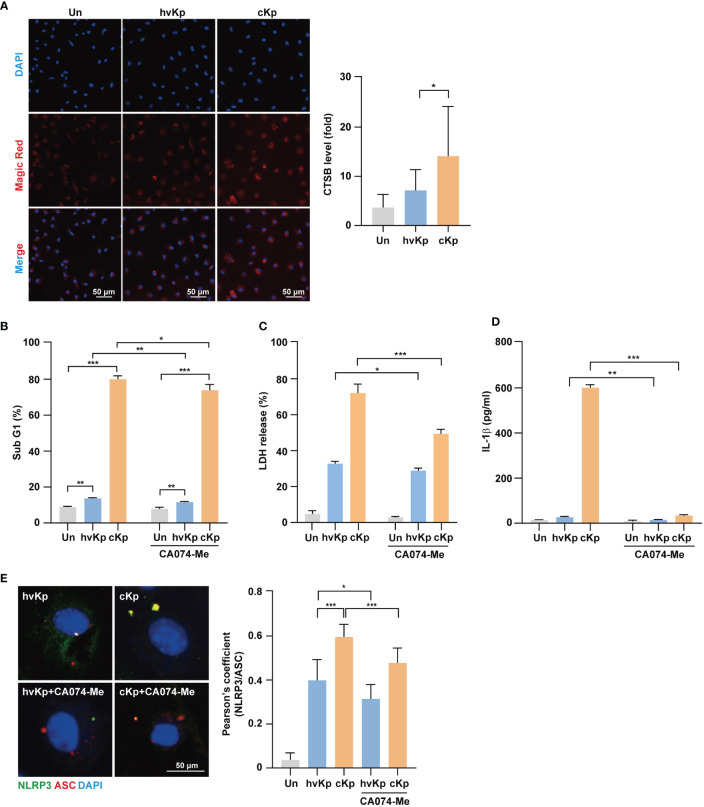
Activation of cathepsin B is involved in pyroptosis induced by hvKp and cKp infections. **(A)** BMDMs were infected with hvKp or cKp (MOI of 10) for 8 h, and cells were stained with Magic Red for 1 h. Nuclei were stained with Hoechst 33342. The images were visualized using confocal microscopy. Scale bar, 50 μm (left). Quantification of cathepsin B activity level (right). **(B)** BMDMs were pretreated with CA074-Me (25 μM) for 1 h, followed by infection with hvKp or cKp (MOI of 10). After incubation for 24 h (hvKp) or 16 h (cKp), cells were stained with PI and subjected to flow cytometry. **(C, D)** BMDMs were incubated in the presence or absence of CA074-Me for 1 h and then infected with the hvKp or cKp (MOI of 10), and after 24 h (hvKp) or 16 h (cKp), the supernatant was collected. **(C)** LDH release was measured in the supernatant and **(D)** IL-1β levels were determined using ELISA. **(E)** BMDMs were incubated in the presence or absence of CA074-Me for 1 h and then infected with hvKp or cKp (MOI of 10) for 8 h. Cells were stained with NLRP3 (green), ASC (red), and DAPI (for nuclei). Scale bar, 50 μm (left). NLRP3 and ASC colocalization were analyzed by calculating Pearson’s correlation coefficient (right). Data are expressed as the mean ± standard deviation of the three independent experiments. *P < 0.05, **P < 0.01 and ***P < 0.001 according to analysis of variance. Un, uninfected; CTSB, cathepsin B.

As lysosomal cathepsin B is involved in NLRP3 inflammasome activation (14, 29), we examined whether cathepsin B activity contributes to the elevated NLRP3 inflammasome formation and pyroptosis during infection. During hvKp and cKp infection, Magic Red, which measures the cathepsin B activity, increased in BMDMs (**Figure 5A**). The cells were stained with LysoTracker, and their fluorescence intensity showed a remarkable reduction in cKp-infected macrophages compared with that in hvKp-infected cells (**Supplementary Figure 2A** for BMDMs and **Supplementary Figure 2B** for RAW264.7 cells); this finding suggests that hvKp and cKp infections induce cathepsin B leakage from lysosomes in macrophages.

Next, we investigated whether increased cathepsin B activity due to infection enhances cell death during infection. BMDMs were infected with hvKp and cKp strains in the presence or absence of CA074-Me, an irreversible cathepsin B inhibitor, and cell death was assessed by fluorescence-activated cell sorting and LDH release assays. During infection, the cytotoxic effect was diminished by cathepsin B inhibition in BMDMs (**Figures 5B, C**) and RAW264.7 cells (**Supplementary Figure 2C**). In cKp-infected macrophages, the cytotoxic effect decreased robustly. Moreover, CA074-Me treatment reduced the production of IL-1β (**Figure 5D**) and caspase-1 activity in macrophages (**Supplementary Figures 2D, E** for BMDMs and **Supplementary Figure 2F** for RAW264.7 cells) during infection. In addition, we examined whether cathepsin B inhibition blocked the formation of NLRP3 inflammasome. As shown in **Figure 5E**, the colocalization between NLRP3 and ASC decreased following CA074-Me treatment. Taken together, these data demonstrate that cathepsin B release from lysosomes contributes to the NLRP3 inflammasome induction, caspase-1 activity, and cell death during hvKp and cKp infections.

## 4 Discussion

Since the first community-onset invasive liver abscess reported in East Asia caused by Kp exhibited a hypervirulent characteristic (30, 31), studies reporting hvKp have dramatically increased worldwide (32). The cKp strain has also been considered as an important pathogen over the last two decades (33) as it has shown carbapenem resistance. Clinically, hvKp infection causes severe inflammatory reactions, induces abscess formation, and is frequently accompanied by metastatic lesions such as endophthalmitis and myositis compared with cKp infection (34). Over time, hvKp strains have become increasingly resistant to antibiotics. The hvKp strains acquire antimicrobial resistance genes by inserting resistance plasmids or resistance elements into the hyKp virulence plasmid (35, 36). These bacterial strains can cause severe infections. However, it remains unknown why hvKp exhibits pathogenicity with severe inflammatory reactions. Therefore, a mechanistic understanding of the immunological responses is warranted to understand the pathogenesis of hvKp infection.

Cell death has a crucial role in aspects of immune responses against pathogen infection. Pyroptosis, a type of regulated cell death, controls microbial survival and contributes to the regulation of the host immune response (37–39). Recent studies of the role of pyroptosis in Kp infection have suggested that Kp-induced pyroptosis is important for the development of host immunity. When HEp-2 cells were treated with OmpA, the transmembrane porin protein of Kp (ATCC 13883), OmpA induced cell cycle arrest, apoptosis, and pyroptosis. These events may act as host defense mechanisms against Kp infection (40). In addition, caspase-11-mediated IL-1α and IL-1β secretion, and pyroptosis were involved in the early stages of Kp infection (41). However, Codo et al. showed that different clinical strains of Kp exhibited opposite behaviors within the macrophages (23). A28006, a Kp carbapenemase-2-producing clinical strain, triggered high levels of pyroptosis and IL-1β production, leading to bacterial clearance via efferocytosis (23). Meanwhile, the A54970 clinical strain inhibited pyroptosis and inflammasome activation through the production of IL-10 (23). We also observed differential levels of pyroptosis between the hvKp and cKp strains in the macrophages. Similar to the A28006 Kp strain, cKp induced high levels of pyroptosis and IL-1β production in murine macrophages. The induction of pyroptosis can trigger pore-induced intracellular traps (PITs), which clear pathogens by efferocytosis and increase the susceptibility to antibiotics and oxidative stress (42). Based on this finding, cKp-infected macrophages may form PITs that clear pathogens and block their dissemination through the induction of pyroptosis.

However, pathogens have evolved counter-strategies to avoid or exploit pyroptosis to maintain survival (43). *Mycobacterium tuberculosis* induces type VII secretion system-dependent plasma membrane damage and pyroptosis, thus allowing *M. tuberculosis* to spread to neighboring cells (26). Li et al. showed that *Shigella flexneri* can escape caspase-11/4-mediated pyroptosis using OspC3, a type III secretion system effector (44). Because Kp also regulates cell death including pyroptosis, it is important to understand cell death caused by Kp infection. A54970 Kp strain inhibited pyroptosis to survive in the host, leading to bacterial dissemination (23). In addition, Kp infection triggered apoptosis and changed cellular ATP levels in HepG2 cells (45). In neutrophil, Kp delayed the induction of apoptosis, which could restrict intracellular bacteria replication (46). Moreover, Kp induced necroptosis of neutrophil, but inhibited apoptosis, resulting in the reduction of efferocytosis (47). Importantly, our study found that the levels of pyroptosis and NLRP3 inflammasome formation were lower in hvKp-infected macrophages compared with those in cKp-infected macrophages. These data suggest that hvKp can control cell death to escape from pyroptosis-induced efferocytosis and PIT formation. Although pyroptosis can be induced by a non-canonical pathway that is activated by the direct interaction between caspase-4/5/11 and cytosolic lipopolysaccharides (9), we focused on investigating the canonical pathway during infection. Notably, we found that the cytotoxic effects were decreased in *Caspase-1* ^-/-^ BMDMs and after caspase-1 inhibitor treatment. These findings demonstrate that hvKp and cKp induce the occurrence of pyroptosis via the activation of caspase-1. We also showed that cytotoxic effects were significantly reduced in hvKp-infected *Caspase-1* ^-/-^ BMDMs, compared with cKp-infected cells.

**Figure 6:**
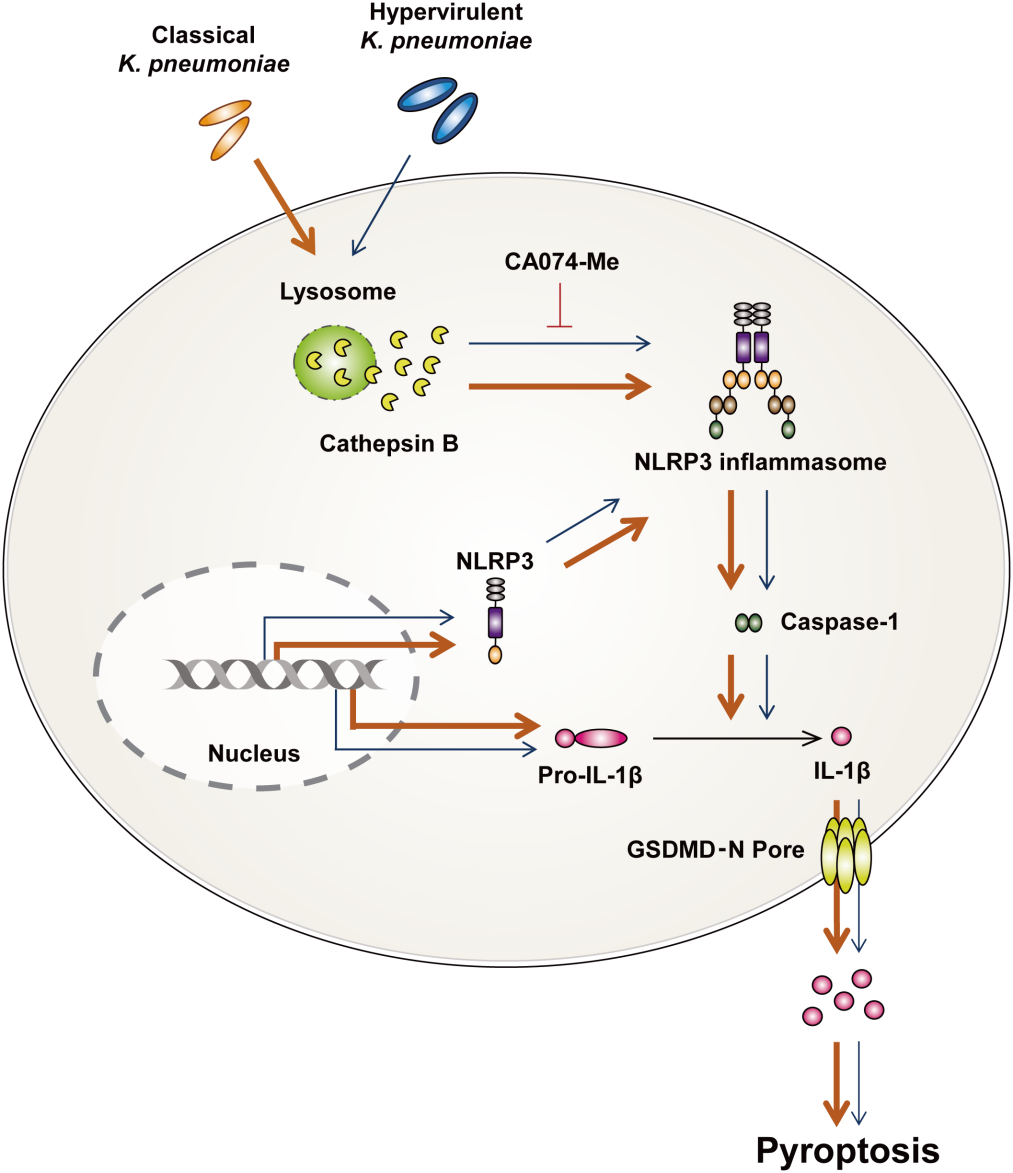
Summarized illustration of hvKp- and cKp-induced pyroptosis in murine macrophages. HvKp and cKp infections induce cathepsin B activation, NLRP3 inflammasome formation, and caspase-1-dependent pyroptosis. All phenomena are inhibited in hvKp-infected macrophages.

To further understand the mechanisms underlying the activation of pyroptosis, we investigated whether cathepsin B influences the occurrence of pyroptosis during infection. Pathogens such as *M. tuberculosis* and *Legionella pneumophila* upregulate the expression and enzymatic activity of cathepsin B (18, 48). During hvKp infection, the transcription of cysteine cathepsin inhibitor was upregulated (49). Additionally, thrombospondin-1-deficient mice showed increased bacterial clearance and survival compared with WT during Kp infection (50). These mice showed enhanced cathepsin G enzymatic activity in neutrophil (50). Although these results suggest that cathepsins can participate in immune responses upon Kp infection, there are no reports that cathepsins are involved in the ability of Kp to survive in macrophages. We found that hvKp and cKp infection increased the enzymatic activity of cathepsin B in murine macrophages. Previous studies have indicated that lysosomal cathepsin B activated pyroptosis through the formation of NLRP3 inflammasome in various cell types (13) and the Kp 43816 strain could trigger pyronecrosis requiring activity of cathepsin B (51). We also found that cathepsin B activation is involved in the induction of pyroptosis and NLRP3 inflammasome formation during infection. The collective findings of this study show that hvKp significantly inhibits induction of cathepsin B-NLRP3-dependent pyroptosis in murine macrophages compared with those in cKp-infected macrophages (**Figure 6**).

The important virulence factors of hvKp were identified. The most frequent contributors to the hypervirulent characteristics are *rmpA* and aerobactin; these virulence genes are associated with a virulence plasmid, regardless of the capsular serotype (52). Since earlier study reported that strains with K1-type capsules delayed apoptosis of neutrophil compared with acapsular mutants (53), we can speculate that the induction of pyroptosis may be delayed in hvKp-infected macrophages. However, further studies are needed to identify the factors that cause these differences in host cells. This study was the first to report the occurrence of pyroptosis during hvKp infection in macrophages. Based on these findings, cathepsin B-mediated pyroptosis is a potential new target for regulating host immune responses during hvKp infection.

## Data availability statement

The original contributions presented in the study are included in the article/**Supplementary Material**. Further inquiries can be directed to the corresponding authors.

## Ethics statement

The animal study was reviewed and approved by Institutional Research and Ethics Committee at Keimyung University.

## Author contributions

JKK and H-JJ performed experiments and wrote the manuscript. MH and JYL collected clinical strains. J-HP provided *Caspase-1* ^-/-^ mice. S-IS supported analytical tools and reagents. W-KB and HAK designed and supervised the study. All authors contributed to the article and approved the submitted version.
