# Supplementary material for: Resistance of hypervirulent Klebsiella pneumoniae to cathepsin B-mediated pyroptosis in murine macrophages
Source: Front Immunol. 2023 Jun 29;14:1207121. doi: 10.3389/fimmu.2023.1207121 (PMC10342201; doi:10.3389/fimmu.2023.1207121)
Supplement: Supplementary file 1 [file DataSheet_1.docx]

Supplementary Material

**Resistance of hypervirulent *Klebsiella pneumoniae* to cathepsin B-mediated pyroptosis in murine macrophages**

**Jin Kyung Kim^*^, Hui-Jung Jung^*^, Miri Hyun, Ji Yeon Lee, Jong-Hwan Park, Seong-Il Suh**

*** Correspondence: Hyun ah Kim**: [hyunah1118@dsmc.or.kr](mailto:hyunah1118@dsmc.or.kr), **Won-Ki Baek:** wonki@dsmc.or.kr

# Supplementary Data

Supplementary Material should be uploaded separately on submission. Please include any supplementary data, figures and/or tables.

Supplementary material is not typeset so please ensure that all information is clearly presented, the appropriate caption is included in the file and not in the manuscript, and that the style conforms to the rest of the article.

# Supplementary Figures and Tables

For more information on Supplementary Material and for details on the different file types accepted, please see [here](https://www.frontiersin.org/guidelines/author-guidelines#supplementary-material).

## Supplementary Figures

**Supplementary Table1.** Characteristics of Kp strains included in this work.

|  | Source | Origin | String Test | Antibiotic Resistance | Serotype | *rmpA* | *magA* | *allS* | *iutA* | *mrkD* | *entB* | *kfu* |
| --- | --- | --- | --- | --- | --- | --- | --- | --- | --- | --- | --- | --- |
| hvKp | Blood | Liver abscessus | (+) | Multi-susceptible | K1 | (+) | (+) | (+) | (+) | (+) | (+) | (+) |
| cKp | Sputum | Pneumonia | (-) | ESBL producer | ND | (-) | (-) | (-) | (-) | (+) | (+) | (-) |

ESBL, extended-spectrum β-lactamase; ND, not determined.


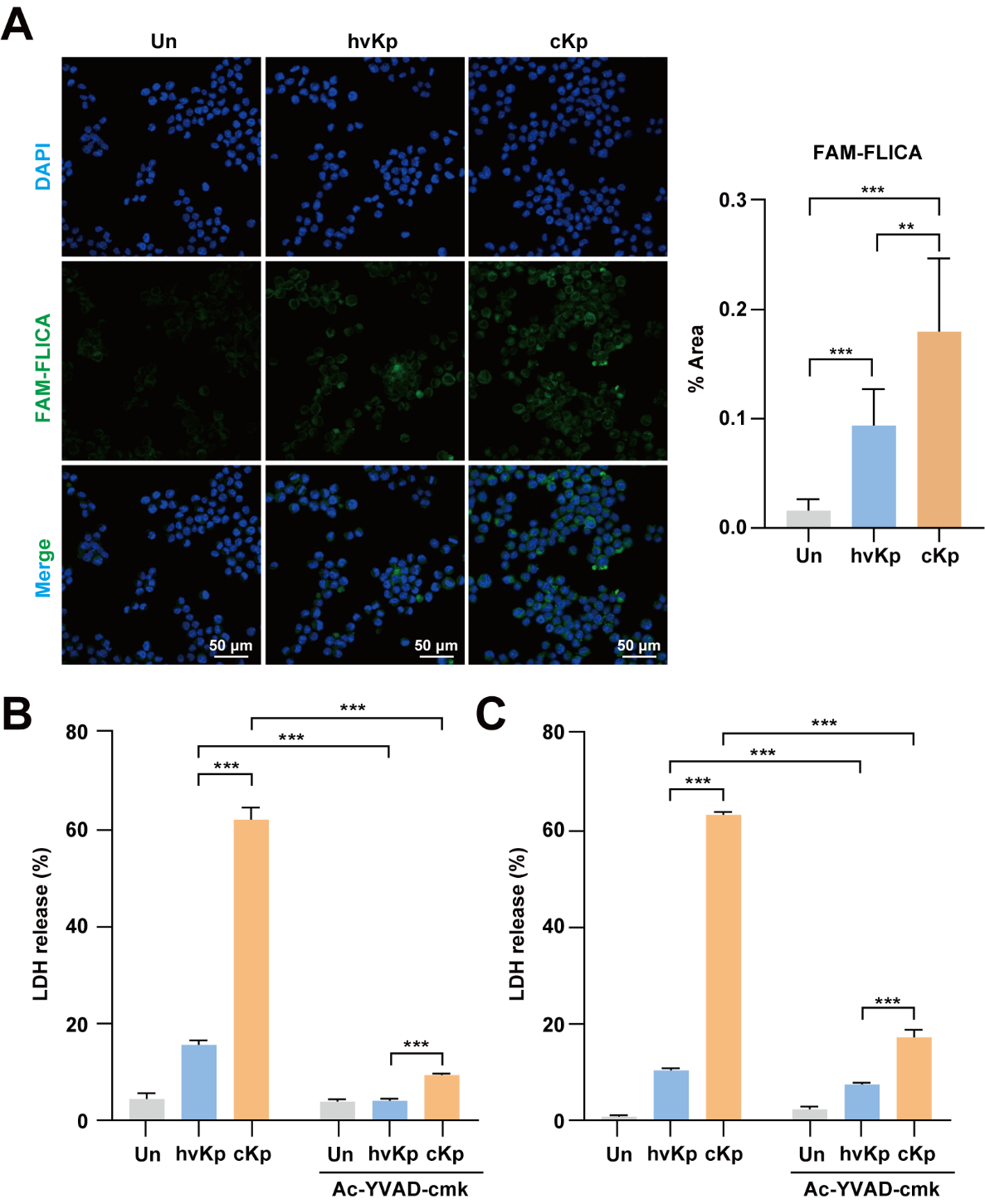


**Supplementary Figure 1.** HvKp and cKp strains induce caspase-1-dependent pyroptosis in macrophages. **(A)** RAW264.7 cells were infected with hvKp or cKp (MOI of 10) for 8 h, and cells were stained with FAM-FLICA for 1 h. Nuclei were stained with Hoechst 33342. The images were visualized by confocal microscopy. Scale bar, 50 μm (left). Quantification of FAM-FLICA area (right). **(B–C)** **(**B**)** BMDMs and **(**C**)** RAW264.7 cells were incubated in the presence or absence of Ac-YVAD-cmk (50 μM) for 30 min, then infected with the hvKp or cKp (MOI of 10) for 16 h. LDH release was measured in the supernatant. Data are express as the mean ± standard deviation or the three independent experiments. **P < 0.01 and ***P < 0.001 according to analysis of variance. Un, uninfected.


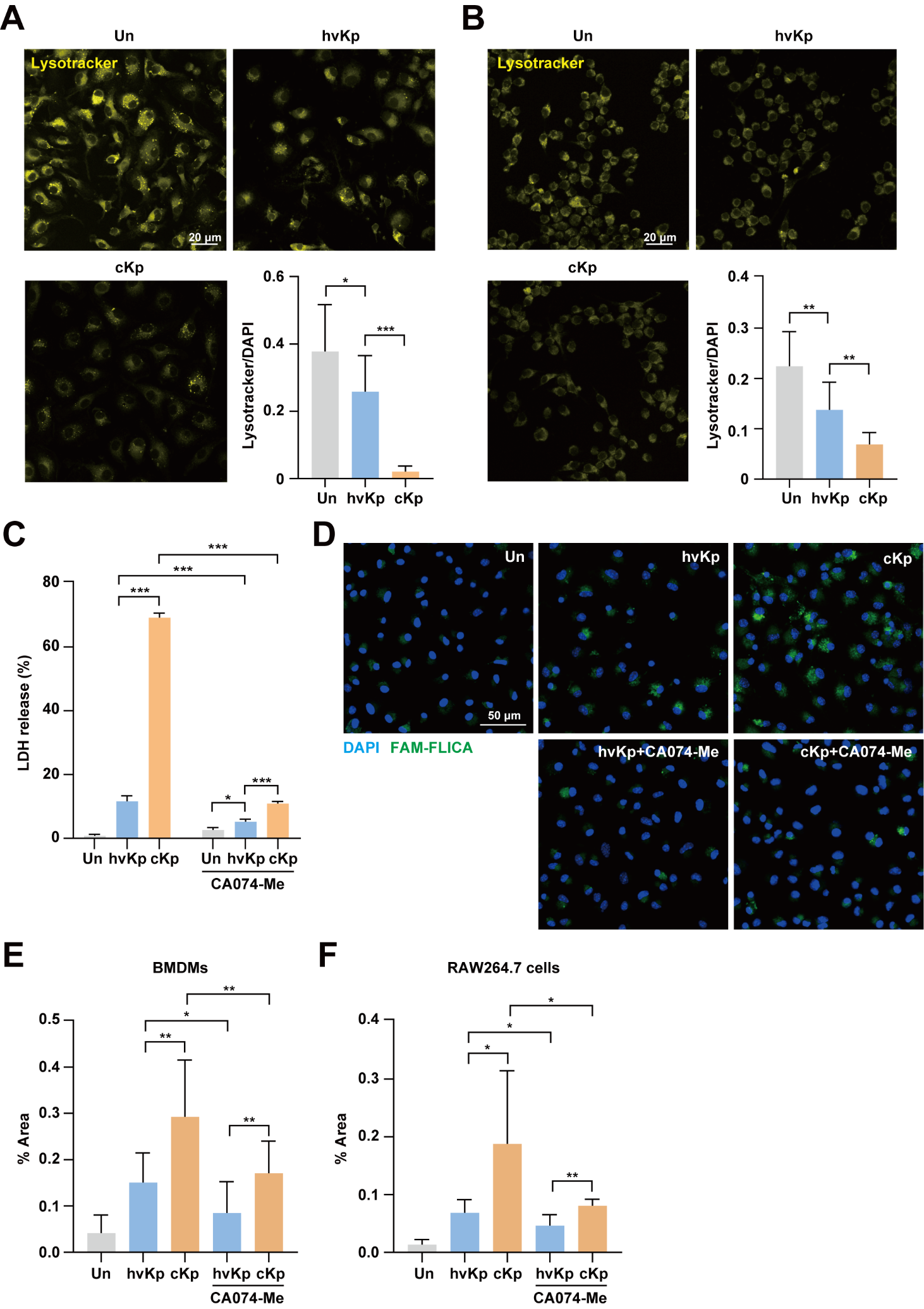


**Supplementary Figure 2.** Cathepsin B from lysosomes is required for induction of pyroptosis and activation of caspase-1 in murine macrophages. **(A, B)** (A) BMDMs or (B) RAW264.7 cells were infected with hvKp or cKp (MOI of 10) for 8 h, and cells were stained with Lysotracker Yellow HCK-123 for 30 min. The images were visualized by confocal microscopy. Scale bar, 20 μm. The quantification of Lysotracker intensity. **(C)** RAW264.7 cells were incubated in the presence or absence of CA074-Me for 1 h and then infected with the hvKp or cKp (MOI of 10). After16 h, LDH release was measured in the supernatant. **(D-F)** (D, E) BMDMs or (F) RAW264.7 cells were incubated in the presence or absence of CA074-Me for 1 h and then infected with hvKp or cKp (MOI of 10) for 8 h. Cells were stained with FAM-FLICA for 1 h. Nuclei were stained with Hoechst 33342. (D) The images were visualized by confocal microscopy. Scale bar, 50 μm. (E, F) Quantification of FAM-FLICA area. Data are express as the mean ± standard deviation or the three independent experiments. *P < 0.05, **P < 0.01 and ***P < 0.001 according to analysis of variance. Un, uninfected.
